# ﻿A new genus and three newly recorded species of Encyrtidae (Hymenoptera, Chalcidoidea) from China

**DOI:** 10.3897/zookeys.1193.116791

**Published:** 2024-02-28

**Authors:** Ning Kang, Hongying Hu, Shuhan Guo, Shungang Luo

**Affiliations:** 1 College of Life Science and Technology, Xinjiang University, Urumqi, Xinjiang 830046, China; 2 Xinjiang Key Laboratory of Biological Resources and Genetic Engineering, Xinjiang University, Urumqi, Xinjiang 830046, China; 3 College of Biological Sciences and Technology, YiLi Normal University, YiLi, Xinjiang 835012, China

**Keywords:** Alpine steppes, *Apteronotusindigus*, encyrtids, new genus, taxonomy, wingless

## Abstract

A new genus and species of Encyrtidae (Hymenoptera: Chalcidoidea), *Apteronotus* Kang, Hu & Luo, **gen. nov.** (type species *A.indigus* Kang, Hu & Luo, **sp. nov.**), associated with insects inhabiting *Oxytropis* spp., and three newly recorded species for China, *Copidosomaclavatum*, *Ericydnusaeneus* and *Tetracnemuskozlovi*, are described from the Altun Mountain Nature Reserve, Xinjiang. Detailed illustrations of all species were included to support the identification and further study.

## ﻿Introduction

Encyrtidae, a large family in Chalcidoidea (Hymenoptera), is characterized by their extensive diversity and cosmopolitan distribution, encompassing over 500 genera and 4700 species worldwide, among them, 128 genera and 483 species recorded from China. Encyrtidae are predominantly parasitoids, targeting a wide range of host taxa primarily within Hemiptera, but also extending to Lepidoptera, Coleoptera, Diptera, and other insect groups, as well as other arthropods, including ticks (Noyes 2019). The majority of Encyrtidae species are endoparasitoids, and a few of them are hyperparasitoids (Trjapitzin 1989). Many species of this family have been utilized in the biological control of crop pests, underscoring the family’s ecological and biogeographical significance.

A critical milestone in the study of Chinese Encyrtidae was achieved by Zhang and Huang (2004), who provided an extensive key to 123 genera. Despite this, knowledge of the biological resources of Encyrtidae in vast areas of China is still limited. The Altun Mountain National Nature Reserve, located in Xinjiang, China, is one of China’s four uninhabited areas, which is characterized by unique extreme environmental conditions such as low temperature, strong winds, and high ultraviolet radiation, as well as complex and diverse habitats like widespread sandy and gravel deserts, wetlands and alpine steppes. Under such environmental conditions, the poorly known species of Encyrtidae need to be investigated comprehensively and urgently to reveal its biodiversity and enrich available data on the family for further study on its adaptation to the extreme environment.

In this context, our study focused on encyrtids collected from 2019 to 2021 in the dominant alpine steppes’ habitats within the Altun Mountain Nature Reserve. This research contributes to the taxonomic understanding of the family by documenting four species across four genera. Notably, it includes one new genus, *Apteronotus* Kang, Hu & Luo, gen. nov., and one new species, *Apteronotusindigus* Kang, Hu & Luo, sp. nov. Additionally, we report three species, *Copidosomaclavatum*, *Ericydnusaeneus* and *Tetracnemuskozlovi*, as new distributional records for China. This work represents a significant step in unraveling the taxonomic and ecological complexities of Encyrtidae in an alpine region that has been historically underrepresented in entomological research.

## ﻿Materials and methods

All the examined specimens were collected by using sweeping nets, yellow pan traps as well as malaise traps in July from 2019 to 2021; yellow pans were left from 8 to 24 hours at each site, and alcohol in the malaise traps was changed every 10 (±5) days to 1 month. The specimens were sorted and immediately preserved in absolute ethanol and stored at -20 °C. Selected specimens of both sexes were slide-mounted and labeled or air-dried and card mounted, and examined under a Nikon SMZ745T stereomicroscope using the available keys (Trjapitzin 1989; Zhang and Huang 2004). Habitus photographs were taken with a Nikon D7000 digital camera connected to a Nikon SMZ25 stereomicroscope. Detailed features of the new species were photographed with a LEO-1430VP scanning electron microscope (SEM), and plates were compiled using Adobe Illustrator CC 2017 software. All specimens were deposited in the Insect Collection of the College of Life Science and Technology, Xinjiang University, Urumqi, Xinjiang, China (**ICXU**).

The taxonomic terminology and abbreviations follow Trjapitzin (1989). The following abbreviations are used in the text: **F1–6**, funicle segment number; **POL**, distance between the posterior ocelli; **OOL**, distance between the eye margin and the adjacent posterior ocellus; **OCL**, distance between the posterior ocellus and the occipital margin; and **T1–7**, tergite segment number.

## ﻿Results

### ﻿Genus Apteronotus

Taxon classificationAnimaliaHymenopteraApteronotidae

Kang, Hu & Luo gen. nov.

BBD16B04-4523-535D-BD7F-4EEDE388AFDD

https://zoobank.org/1D48FE0E-4167-426D-87EF-192D33A4A6C1

Figs 1 , 2

#### Type species.

*Apteronotusindigus* Kang, Hu & Luo, sp. nov.

#### Etymology.

Female, “Apteron” refers to lack of wings in this genus, and “notus” is a suffix often used in insect taxonomy.

#### Diagnosis.

This genus exhibits distinct morphological divergences when compared to the two subfamilies (Encyrtinae and Tetracneminae) in Encyrtidae. The new genus can be differentiated from other related genera by a combination of the following characteristics: Body length 0.65–0.75 mm, short and robust, body indigo blue, eyes and ocelli dark red, mandible yellow, tibia and trochanter yellow, basitarsus, and apical tibiae yellow. Head in dorsal view without occipital margin; antenna slender, slightly longer than head width, clava 3-segmented; mesoscutum slightly shorter than head width, with faint reticulation and sparse setose; notaular lines absent, axillae separate apically; propodeum shorter than 1/2 scutellum medially; wings absent in both sexes; mid tibial spur shorter than basitarsus; gaster ovate, posterior margin of T1 medially incised in some individuals, ovipositor sheath not exserted, paratergite not present.

#### Distribution.

China (Xinjiang).

#### Hosts.

Unknown.

#### Comments.

The genus does not run to any genus in the keys (Trjapitzin 1989; Noyes et al. 1997). Extensive morphological comparisons were made with several brachypterous genera (Trjapitzin and Gordh 1979); however, *Globulencyrtus* Hoffer, 1976 differs in the following characteristics: head with sharp occipital margin, clava shorter than funicle, forewing rudiments, and reaching to about apex of scutellum posteriorly (Hayat et al. 2013); *Austrochoreia* Girault, 1929 is distinguished by the elongate pronotum that almost covers the mesoscutum, lack of notaular lines and abbreviated wings (Noyes and Hayat 1984); it can be distinguished from *Aglyptus* (Tetracneminae) by several key characteristics: body chocolate-brown-yellow, with light green shine, female body length 1.9–2.0 mm, forewing not developed and with dark transverse band in the apical third; similarly, the related genus *Bactritopus* with large and deep depression on face, clypeal margin forming a spatulate protrusion, antennal toruli located at the edge of mouth, mandible tridentate, with a long middle tooth, mesoscutum with complete notauli, wings not shortened (Trjapitzin 1978). The specimens also share some characteristics with the genus *Choreia* (Encyrtinae), but they differ notably in having a large punctation on the vertex and frons, occipital margin sharp, mesoscutum usually 3× as broad as long, scutellum roundish in back view, axillae meeting, and female body length at least 1 mm (Westwood 1833; Förster 1856). We also found some characteristic differences within the new species, the hind margin of T1 slightly incised medially in some individuals.

### ﻿ Apteronotus indigus

Taxon classificationAnimaliaHymenopteraApteronotidae

Kang, Hu & Luo sp. nov.

4426FBE7-441B-543E-B66B-0E562CBB242F

https://zoobank.org/8D8E6AD7-86B6-4DE9-ADB6-758CEAA683EF

Figs 1A–H , 2A–F

#### Type material.

***Holotype***, ♀, card mounted, China, Xinjiang, Ruoqiang County, Altun Mountain Nature Reserve, 36°58′10″N, 90°14′45″E, Altitude: 4021.95 m, 14.VII.2020, Coll. Shun-Gang Luo, Ning Kang, Hong-Ying Hu by yellow pan trapping. ***Paratypes*.** 1♀, 1♂, on slide, same data as holotype except 18.VII.2020; 3♀♀, 5♂♂, card mounted, 21.VII.2020. Coll. Shun-Gang Luo, Ning Kang, Hong-Ying Hu (all deposited in ICXU).

#### Description.

**Figure 1.**
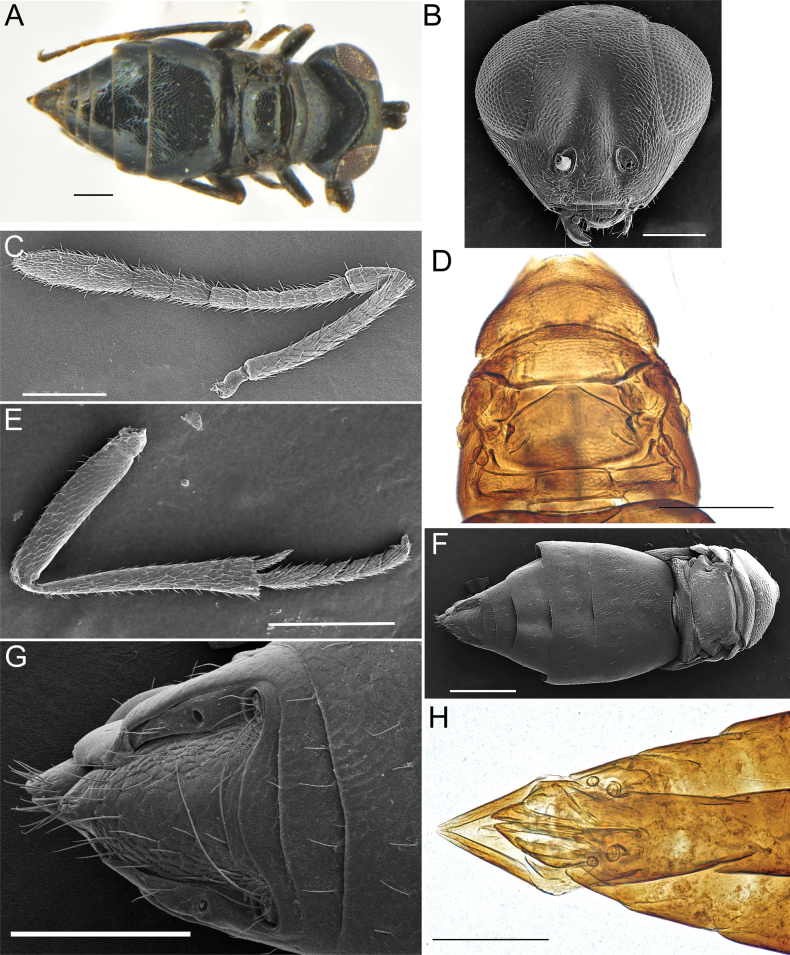
*Apteronotusindigus* Kang, Hu & Luo, sp. nov., female **A** habitus, dorsal view **B** head, frontal view **C** antenna **D** mesoscutum, dorsal view **E** mid leg **F** body, dorsal view **G, H** tip of gaster. Scale bars: 100 μm.

**Figure 2.**
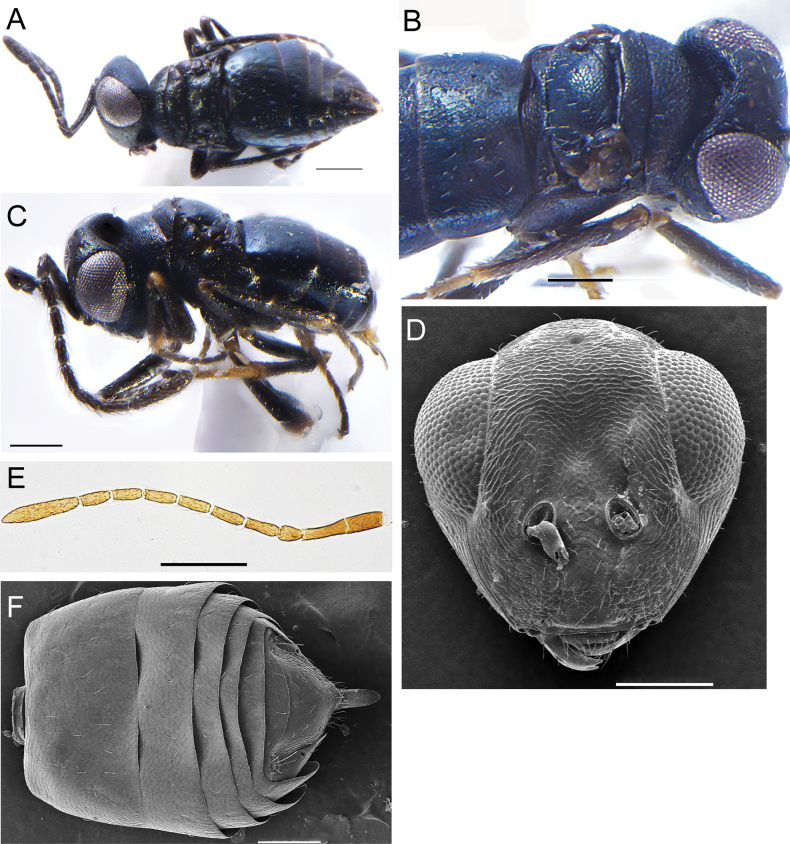
*Apteronotusindigus* Kang, Hu & Luo, sp. nov., male **A** habitus, dorsal view **B** mesoscutum, lateral view **C** habitus, lateral view **D** head, frontal view **E** antenna **F** gaster. Scale bars: 100 μm.

**Female.** Length 0.7 mm. Body black with dark bluish metallic sheen (Fig. 1A). Antenna, femora, and tibiae dark brown, head in dorsal view with bronze shine at some angle. Mesosoma black. Legs with all coxae black; trochanter and their apices deep yellow, tarsal segments 1–3 yellow, fourth and fifth tarsi dark brown (Fig. 2C). Gaster black except T6–7 dark brown.

***Head*** in frontal view 1.2× as broad as high (385:330) (Fig. 1B), 1.9× as broad as mesosoma, with fine reticulate sculpture, covered with sparse and short setae; frontovertex width 0.5× head width (117:385), antenna located below the lower eye margin and separated from clypeus margin by 1.4× height of the torulus, inner edge of eyes diverged at the lower part, malar sulcus distinct and straight, malar space 0.65× as long as eye height (120:185), mandible bidentate. Head in dorsal view 2.1× as broad as long (347:168), ocelli forming an obtuse angle (110°), POL 1.8× as long as OOL (55:30), posterior edge of eye adjacent to posterior margin of head. Antenna with scape cylindrical and covered with short setae, 9× as long as wide (260:28), pedicel 2× as long as wide (60:30); all funicular segments longer than wide (Fig. 1C), each funicular with three rows of irregular longitudinal sensilla, F1 and F3 2× as long as broad respectively (40:20), obviously shorter than pedicel and other segments, F4–F6 subequal in length, 1.5× as long as broad (45:30); clava 3-segmented, 3.6× as long as broad (145:40), longer than the combined length of F5–F6, with obscure boundaries and apically rounded, the collective length of pedicel and flagellum 1.22× as long as head width (470:385).

***Mesosoma*** much shorter than metasoma and reticulate sculpture larger than that on head. Pronotum 1.8× as long as mesoscutum length (90:50) and 2.78× as broad as long (250:90); mesoscutum 5.2× as wide as long (260:50) (Fig. 1D), notaular lines absent, tegula large and semicircular; scutellum slightly convex and 2.2× as wide as long (250:117), scattered with some short white setae irregularly arranged on its posterior part, axillae very small and apically separated; propodeum medially longer than 1/3 of the scutellum (40:117), with sinuated inverted U-shaped ridges (Fig. 1F). Mesotibial spur strong, 0.6× as long as basitarsus (63:104) (Fig. 1E). Wings absent.

***Gaster*** 1.6× as long as broad (694:426), longer than the combined length of head and mesosoma, T1 distinctly longer than other tergites individually (250:444), occupying about two-fifths of the total length, covered with 3–4 rows of setae, T2–T6 each with a single row of setae, T7 with 2–3 rows of short setae, and with 5 long cercal bristles at each side. Paratergites absent. Hypopygium slightly extends to the apex of gaster (Fig. 1G, H).

**Male.** Length 0.57–0.64 mm (0.6±0.042 mm, *N* = 5) (Fig. 2A–C), similar to female in body color and sculpture, but differs as follows. Frontovertex slightly arched (Fig. 2D); each funicular segment clearly elongated, 3× as long as broad, with at least 2 rows of long black whorled setae (Fig. 2E); pedicel distinctly shorter than F1, 0.67× as its length; clava 4.25× as long as broad; the combined length of pedicel and flagellum 1.6× as long as head width. Gaster 1.3× as long as broad, posterior margin of each tergite straight (Fig. 2F).

#### Hosts.

Unknown.

#### Etymology.

“*indigus*” means indigo blue, signifying the body color of the female species.

### ﻿Genus Copidosoma

Taxon classificationAnimaliaHymenopteraEncyrtidae

Ratzeburg, 1844

69778982-1A9A-5545-BC44-C83E3058D25D

#### Note.

The genus is widely distributed worldwide, with 204 valid species, 22 of which have been recorded from China (Zhang and Huang 2007). Most species of the genus are parasitic on lepidopteran insects, especially endoparasitic in eggs and larvae (Noyes 2019), which provide good prevention and control effects on agriculture and forestry pest populations.

### ﻿ Copidosoma clavatum

Taxon classificationAnimaliaHymenopteraEncyrtidae

Myartseva, 1982

105771E6-ADDF-5C80-9ED1-C08590ADA2A5

Fig. 3A–G

Copidosoma clavatum Myartseva, 1982: 26.

#### Material examined.

China: 3♀♀, card mounted, Xinjiang, Ruoqiang, Altun Mountain Nature Reserve, 37°58′30.15″N, 88°58′25.15″E, Altitude: 3489 m, 14.VII.2020. Coll. Shun-Gang Luo; 2♀♀, 36°58′10.89″N, 90°14′44.19″E, Altitude: 4021.95 m, 21.VII.2020. Coll. Shun-Gang Luo, by yellow pan trapping (all depo­sited in ICXU).

#### Diagnosis.

**Figure 3.**
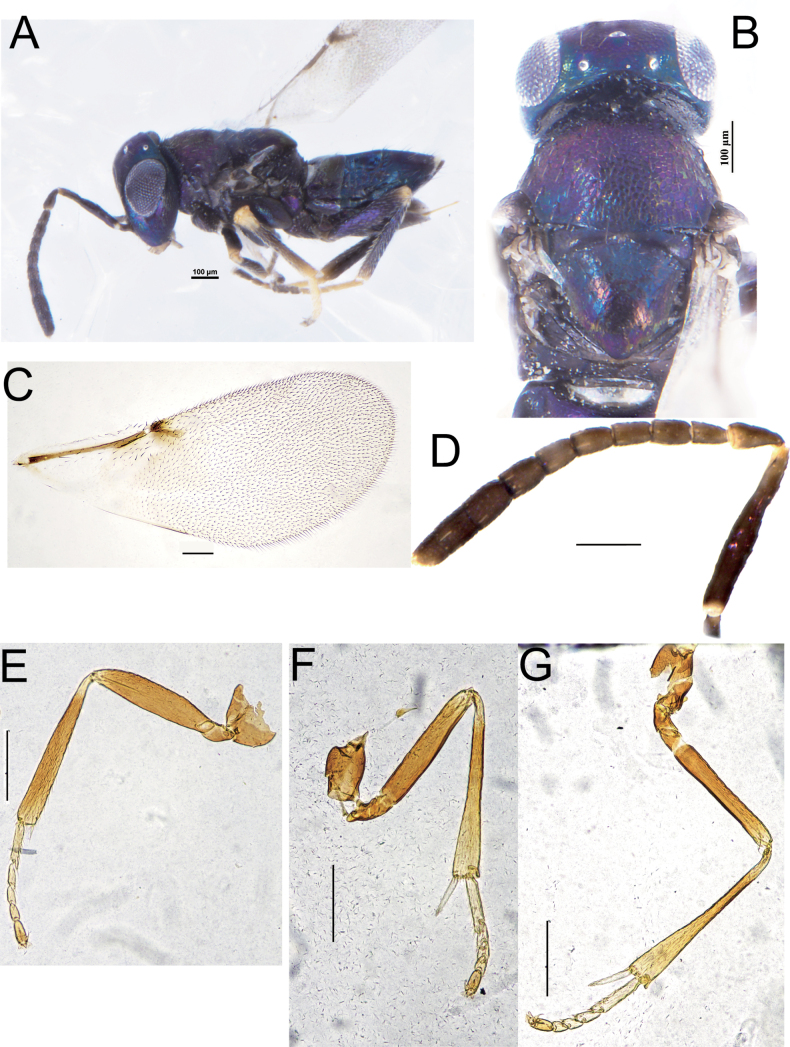
*Copidosomaclavatum* Myartseva, female **A** habitus, lateral view **B** head and mesoscutum, dorsal view **C** forewing **D** antenna **E** fore leg **F** mid leg **G** hind leg. Scale bars: 100 μm.

**Female**. Length 1.12–1.45 mm (Fig. 3A), body ink blue, with blue-purple metallic luster; ocelli silver, eyes argenteous; antenna and leg dark brown; tibiae concolorous with body, trochanter, distal tibiae and tarsi yellow (Fig. 3E–G); forewing hyaline, venation dark brown. Head width equal to its height, ocelli forming an obtuse angle range from 96–105°, OOL about 1.55× OCL, torulus below the ventral margin of eye, F1–F6 equal in length, gradually widen towards the end, scape less than 6× as long as width, clava 3-segmented, with slight oblique truncation (Fig. 3D); mandible tridentate, with median tooth longest. Mesoscutum with honeycomb reticulate, axillae separated apically (Fig. 3B); linea calva complete, postmarginal vein punctate (Fig. 3C); mesotibial spur 0.87× basitarsus (Fig. 3F). The exerted ovipositor obviously shorter than mesotarsus.

**Male**. Unknown.

#### Hosts.

Unknown.

#### Distribution.

China (Xinjiang) new record; India (Andhra Pradesh, Kerala, Odisha, Tamil Nadu, West Bengal) (Kazmi and Hayat 2012), Turkmenistan (Trjapitzin 1989).

#### Comments.

The similar species *C.aretas* can be separated from this species by the body color dark green, funicle elongated distally, scape more than 6× as long as width, clava without oblique truncation, and forewing linea calva interrupted posteriorly (Trjapitzin 1989).

### ﻿Genus Ericydnus

Taxon classificationAnimaliaHymenopteraEncyrtidae

Haliday, 1832

3539C44D-AC81-5F11-8AF4-C5112EA95CE1

#### Note.

The genus has 33 valid species in the world, mostly distributed in the Palearctic region with 11 species from China. The distinct characteristics of this genus are mandible bidentate, all funicles longer than wide; mesoscutum covered with white setae, scutellum with membranous sharp flange apically, and overhanding propodeum; forewing infuscate, linea calva complete, veins long, stigmal with long uncus.

### ﻿ Ericydnus aeneus

Taxon classificationAnimaliaHymenopteraEncyrtidae

Nikolskaya, 1952

94BEF784-BA70-5033-83E9-7C086454EE89

Fig. 4A–G

Ericydnus aeneus Nikolskaya, 1952: 357.
Ericydnus (Aeneus) robustior Nikolskaya, 1952: 96. Synonymized by Trjapitzin 1989.

#### Material examined.

5♀♀8♂♂, China: Xinjiang, Ruoqiang, Altun Mountain Nature Reserve, 36°58′10.89″N, 90°14′44.19″E, Altitude: 4021.95 m, 14–21.VII.2020. Coll. Shun-Gang Luo, by malaise trap; 4♀♀3♂♂, 37°51′49.39″N, 89°36′31.77″E, Altitude: 3782 m, 13.VII.2020. Coll. Shun-Gang Luo, by yellow pan trapping (all deposited in ICXU).

#### Diagnosis.

**Figures 4.**
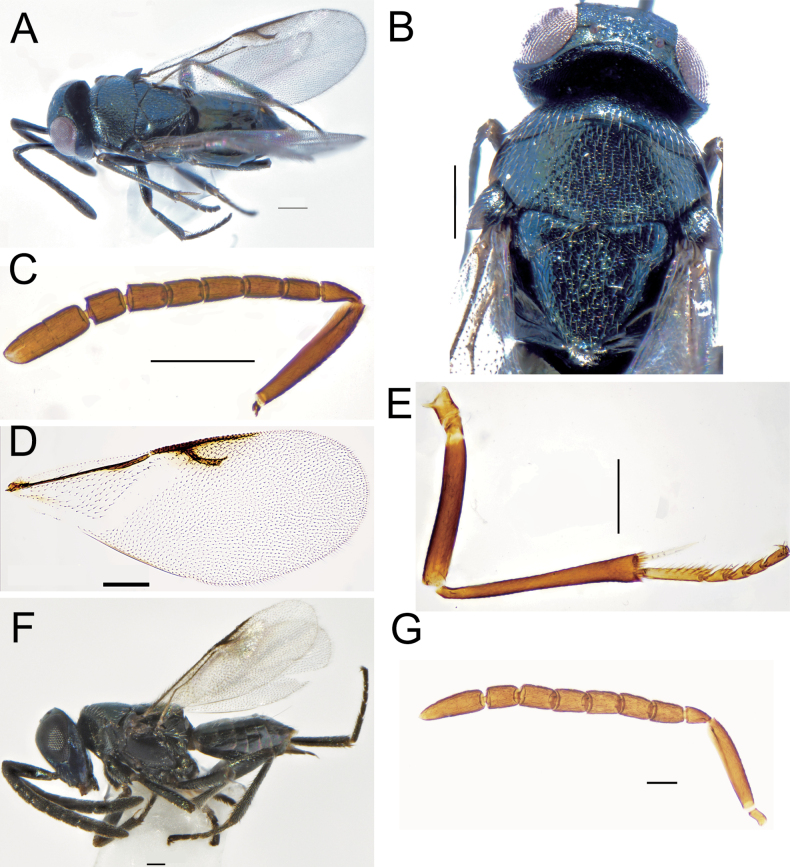
*Ericydnusaeneus* Nikolskaya, female **A** habitus, dorsal view **B** mesoscutum, dorsal view **C** antenna **D** forewing **E** mid leg. Male: **F** habitus, dorsal view **G** antenna. Scale bars: 100 μm.

**Female**. Length 1.65–1.95 mm (Fig. 4A); body dark aeneous, head and mesoscutum covered with distinct white setae, antenna and legs black, eyes and ocelli dark red; forewing hyaline, with infuscate around postmarginal and stigmal vein, venation yellow-brown (Fig. 4D); tarsi dark brown. Head width around 1.09× head length in frontal view, with light reticulate, ocelli forming an obtuse angle (103–107°), OCL 0.82× OOL; torulus below the level of lower eye margin, F1–F6 shortened towards the end, clava 3-segmented, apex with oblique truncation (Fig. 4C), mandible bidentate. Mesoscutum flat, axillae touching (Fig. 4B); forewing hyaline, with infuscate mark under postmarginal and stigmal vein, linea calva entire; mid tibia slightly shorter than exerted ovipositor, its spur slightly longer than basitarsus (Fig. 4E). The exerted hypopygium 0.14× as long as gaster.

**Male.** Length 1.35–1.52 mm (Fig. 4F), clava unsegmented (Fig. 4G), apical rounded, other characters same as female.

#### Hosts.

Pseudococcidae, *Trionymusperrisii* and *Trionymusmultivorus* (Japoshvili and Hansen 2013).

#### Distribution.

China (Xinjiang) new record; Azerbaijan, Europe, Norway, Portugal, Romania, Spain, Turkey, Uzbekistan.

#### Comments.

*Ericydnusdanatensis* is similar to *E.aeneus* but differs in the following characters: head 1.5× as wide as long, torulus at the level of lower eye margin, forewing with distinct dark bands (Myartseva 1980; Sharkov 1986).

### ﻿Genus Tetracnemus

Taxon classificationAnimaliaHymenopteraEncyrtidae

Westwood, 1837

3E4B8E7B-3C59-5D84-8651-AC4761084CC7

#### Note.

The most important characteristics of the genus are the wide and flat flagellum, clava only one segment, and mandible bidentate. The genus encompasses 36 species worldwide, with three species recorded in China. These species are widely distributed throughout the world and their dominant hosts belong to Pseudococcidae (Noyes 2019).

### ﻿ Tetracnemus kozlovi

Taxon classificationAnimaliaHymenopteraEncyrtidae

Sharkov, 1984

508D43B3-7418-532D-B21C-1BFB21EE100D

Fig. 5A–G

Tetracnemus kozlovi Sharkov, 1984: 90–91.

#### Material examined.

10♀♀28♂♂, China, Xinjiang, Ruoqiang, Altun Mountain Nature Reserve, 36°56′25.85″N, 90°16′48.23″E, Altitude: 4023 m, 21.VII.2020. Coll. Shun-Gang Luo, by yellow pan trapping. 8♀7♂, 36°58′10.89″N, 90°14′44.19″E, Altitude: 4021.95 m, 21.VII.2021. Coll. Ning Kang, by sweeping (all deposited in ICXU).

#### Diagnosis.

**Figures 5.**
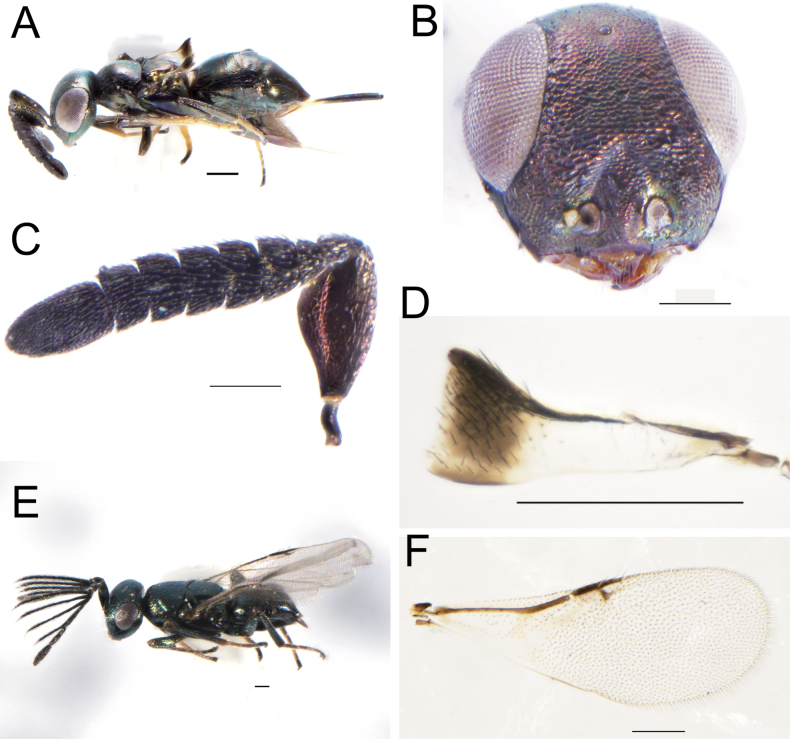
*Tetracnemuskozlovi* Sharkov, female **A** habitus, lateral view **B** head, frontal view **C** antenna **D** forewing. Male: **E** habitus, dorsal view **F** forewing. Scale bars: 100 μm.

**Female.** Length 1.5–2 mm, body deep green with purple metallic luster at mesoscutum (Fig. 5A); eyes dark red, mandible yellowish-brown; antenna and legs dark brown, scape with metallic reflection; wings with basal 2/3 hyaline and tip 1/3 with dark band; the distal 1/2 of tibiae and tarsi yellow. Torulus below the ventral edge of eyes (Fig. 5B); scape distinctly enlarged ventrally, flagellum obviously widened (Fig. 5C), ocelli forming an acute angle (75–85°), OCL 2.15× OOL; scape about 2× as long as broad, and ventral side enlarged obviously, all the funicles transverse, clava unsegmented. Mesoscutum with shallow reticulate engraving, axillae separated medially, wings degenerated, not exceeding the propodeum and truncated distally (Fig. 5D). The exserted part of ovipositor 0.6× gaster length.

**Male.** Length 1.2–1.3 mm (Fig. 5E), antennal funicle with long branches, clava unsegmented; forewing not degenerated and length exceeding beyond the end of gaster (Fig. 5F), wings infuscate, venation brown, linea calva interrupted with four lines of seta, post marginal vein shorter than marginal vein, longer than stigmal vein.

#### Hosts.

Unknown.

#### Distribution.

China (Xinjiang) new record; Russia.

#### Comments.

For the similar short-winged species within the genus, there are obvious morphological differences compared to this species. For example, the antennal scape of *T.subapterus* is not broadened or flattened, each funicle segment is longer than wide, and the ovipositor sheaths are very short. The base of the antennal scape in *T.hofferi* is noticeably shortened, ovipositor sheaths are about 2/3 length of gaster, and the head has a deep microcellular sculpture. The antennal scape of *T.heydeni* is smoothly rounded ventrally, ocelli form an equilateral triangle, and the outer edges of the scrobes are acute (Trjapitzin 2012).

## Supplementary Material

XML Treatment for Apteronotus

XML Treatment for Apteronotus indigus

XML Treatment for Copidosoma

XML Treatment for Copidosoma clavatum

XML Treatment for Ericydnus

XML Treatment for Ericydnus aeneus

XML Treatment for Tetracnemus

XML Treatment for Tetracnemus kozlovi

## ﻿Additional information

### Author ORCIDs

Ning Kang https://orcid.org/0000-0002-9616-6886

Hongying Hu https://orcid.org/0000-0002-2295-1072

### Data availability

All of the data that support the findings of this study are available in the main text.
